# Physiological roles of propolis and red ginseng nanoplatforms in alleviating dexamethasone-induced male reproductive challenges in a rat model

**DOI:** 10.1007/s11033-023-08991-4

**Published:** 2024-01-04

**Authors:** Rabie L. Abdel Aziz, Ahmed Abdel-Wahab, Abdel-Razik H. Abdel-Razik, Shaimaa Kamel, Ahmed A. Farghali, Romaissaa Saleh, Rehab Mahmoud, Marwa A. Ibrahim, Taghred M. Nabil, Fatma I. Abo El-Ela

**Affiliations:** 1 Department of Theriogenology, Faculty of Veterinary Medicine, Beni-Suef University, Beni-Suef, 62511 Egypt; 2 Physiology Department, Faculty of Veterinary Medicine, Minia University, El-Minia, 61519 Egypt; 3 Department of Histology, Faculty of Veterinary Medicine, Beni-Suef University, Beni- Suef, 62512 Egypt; 4 Department of Biochemistry and Molecular Biology, Faculty of Veterinary Medicine, Cairo University, Giza, Egypt; 5 Materials Science and Nanotechnology Department, Faculty of Postgraduate Studies for Advanced Sciences, Beni-Suef University, Beni-Suef, Egypt; 6 Department of Chemistry, Faculty of Science, Beni-Suef University, Beni-Suef, 62511 Egypt; 7 Department of Pharmacology, Faculty of Veterinary Medicine, Beni-Suef University, Beni-Suef, 62511 Egypt

**Keywords:** Propolis, Ginseng, Nanosynthesis, Antioxidant, Genes, Reproduction, Rat

## Abstract

**Background:**

Red ginseng and propolis are well-known antioxidants that have been related to a reduction in oxidative stress.

**Objective:**

This study evaluated the efficiency of red ginseng and propolis, either in powder or as nano-forms against dexamethasone—induced testicular oxidative challenges in adult male albino rats.

**Methods:**

Forty rats were divided into 8 equal groups including control negative group that was given vehicle (DMSO), control positive group that was administered dexamethasone in addition to the nano-propolis, nano-ginseng, nano-propolis + dexamethasone, nano ginseng+dexamethasone, propolis+dexamethasone and ginseng + dexamethasone groups. Serum, semen and tissue samples were obtained.

**Results:**

Lower testosterone levels, higher levels of MDA, and lower levels of total antioxidant capacity in serum, as well as impaired semen quality and a disturbed histopathological picture of both the testis and seminal glands, were all observed as significant negative effects of dexamethasone. These findings were confirmed by lower gene expression profiles of CYP11A1, *StAR*, HSD-3b, *Nrf-2* and *ACTB*-3b in testicular and seminal gland tissues. The most powerful anti-dexamethasone effects were obtained with either propolis in nanoform or conventional ginseng.

**Conclusion:**

Propolis nano-formulation and ginseng in conventional form could be considered excellent candidates to ameliorate the oxidative stress provoked by dexamethasone, however, neither nano-ginseng nor conventional propolis showed such effects.

**Supplementary Information:**

The online version contains supplementary material available at 10.1007/s11033-023-08991-4.

## Introduction

The male reproductive system is one of the most sensitive systems liable to oxidative stress provoked by exposure to environmental insults in different species [1]. Effects of oxidative stress on male reproductive function are mediated through different pathways leading to increased pro-oxidant levels such as malondialdehyde (MDA) and thiobarbituric acid reacting substances (TBARS) [2]; abnormal DNA methylation patterns [1, 3], and abnormal spermatogenesis [4].

Glucocorticoids such as dexamethasone have been included in treatment protocols for several health problems in humans and different animal species. Their inevitable side effects on male reproductive function are still under investigation, particularly, in extended treatment protocols. Of these effects, significant decreases in body weight gains and testicular weight, altered redox status, reduced circulating concentrations of testosterone and thyroid profiles and increased oxidative stress in testicular tissues have been observed [3, 5] in addition to greater risks of peroxidation-mediated testicular damage [6]. Other studies reported atrophy of testicular seminiferous tubules, lipid peroxidation and enhanced apoptosis in spermatocytes following dexamethasone administrations [7]. The adverse effects have been extended to hypothalamic-hypophyseal-gonadal axis dysfunction as documented by Leroy et al. [8]. Annie et al. [9] revealed that dexamethasone treatment of Swiss albino mice was associated with a significant reduction of testosterone levels, and down-regulation of PGC1-α and visfatin which are considered as principal key regulators of steroidogenesis in male animals. The authors also observed that dexamethasone treatment was accompanied by significant reductions in antioxidant enzyme activities in testis. In stallions, an elegant study by Ing et al. [10] showed that dexamethasone when administered to mature stallions resulted in a 94% reduction of serum testosterone from 4 to 20 h. after the initial injection. Collectively, dexamethasone as a treatment has several side effects on male reproductive function in different species including humans. Yet, its precious therapeutic impacts necessitate investigating novel approaches to alleviate such negative effects.

Red ginseng and propolis are known for their potential antioxidant activities and have been repeatedly cited to ameliorate oxidative stress provoked by various insults in the male reproductive system and other body systems [11]. Despite that, some issues still represent an obstacle against their therapeutic applications such as poor water solubility, a limited formulation that can be administered by oral or parenteral routes, undesired organoleptic properties and instability [12]. The application of nanotechnology in therapeutic drug delivery is a well-established technology that conveys several beneficial results via reduced particle size and increased surface area which lead to improved drug absorption and bioavailability and release of functional ingredients, including propolis and ginseng [13].

Ginseng was an excellent candidate to counteract the adverse effects of dexamethasone on hepatic function via switching the oxidant status and apoptotic pathways from deviation to normal state [14]. Moreover, the reproductive performance that was impaired markedly with Ketoconazole (antifungal therapy) was observed to be significantly improved by ginseng [15]. In addition, treating rats with propolis was effective in enhancing the testicular activity that was sharply deteriorated with copper. These improving effects were achieved by upgrading the sperm quality, enriching the testicular tissues with the enzymatic antioxidants together with eliminating of the pro-oxidants (malondialdehyde) and fixing the histopathological deviations [16].

Effects of ginseng and propolis either in powder form or as nano-formulations on dexamethasone-induced reproductive dysfunction have been investigated in a limited number of studies to the best of our knowledge. Thus, we designed this study to compare the efficacy of the two natural compounds either as conventional powdered forms or nano-formulations in ameliorating the reproductive toxicity induced by dexamethasone in adult male albino rats.

## Materials and methods

### Animals

The experiment was carried out using 40 adult male albino rats which were divided into 8 equal groups. Rats were obtained from the lab animal unit, department of Pharmacology, Faculty of Veterinary Medicine, Cairo University, Egypt. After arrival, rats were acclimatized to the basal rodent diet for 15 days before induction of experimental procedures. Rats were fed a standard rodent diet *ad* libitum and received clean fresh water available 24 h. All animal handling procedures were carried out following the protocol approved by the institutional animal care and use committee, Faculty of Veterinary Medicine, Beni-Suef University, Egypt (Approval number: 022–224). A standard 12 h light and dark system was applied during the acclimation and the experimental periods.

### Chemicals

Dexamethasone was purchased from a local pharmacy (Dexamethasone ampule 2 ml capacity containing 8 mg dexamethasone sodium phosphate, Amriya Pharmaceuticals, Egypt). Propolis and ginseng were purchased from a local Market as raw plants. They were identified by experts in the lab of Materials Science and Nanotechnology Department, Faculty of Postgraduate Studies for Advanced Sciences, Beni-Suef University, Egypt. Ginseng and propolis powders including those of nano-particles were dissolved in DMSO 1%. All tested materials in normal or nano-form were administered orally except for dexamethasone which was injected subcutaneously.

### Synthesis of nano-propolis and nano-ginseng

Nano propolis and nano ginseng were obtained through a top-down synthesis approach. The raw propolis and the raw ginseng were subjected to a size-reduction process using the ball milling technique. Ball milling is a widely used top-down nanoparticle synthesis approach involved in many studies [17, 18]. The commercial propolis or ginseng was inserted into a photon ball stainless steel milling vessel. The used balls were fabricated from porcelain and the ball/natural zeolite mass ratio was 10:1. Ball diameter ranged from 1.5 to 1.8 cm, and vessel diameter was 7.5 cm for 24 (10) h with a continuous rotational speed of 200 rpm.

### Characterizations of nano-propolis and nano-ginseng

The phase formation and crystallinity of the nanoparticles were characterized by XRD technique. The vibrations of the material chemical bonds were examined by Fourier Transform Infra-red (FT-IR, Bruker Vertex 70). The morphology analysis of used materials was characterized by a Scanning Electron Microscope (FESEM, Quanta FEG 250). The hydrodynamic size and zeta potential that were provided in supplementary data (Supplementary Table 1 as well as Supplementary Figs. 2, 3 and 4) were studied (experimentally optimized) by Malvern (Malvern Instruments Ltd), using the methodology mentioned previously [19].

### Experimental procedures

The experiment was carried out for one month on 40 adult male albino rats weighing 150–200 gm body weight and 2–3 months age. Rats were divided into 8 equal groups as follows:

Group one (Control negative), received DMSO 1% in sterile distilled water (0.5 ml) once daily by stomach gavage throughout the experimental period. In group two (Dexamethasone), each rat was administered with dexamethasone, thrice-weekly by SC injection of 0.1 ml (7 mg/kg b.wt.), as a control positive group according to Hasona [5]. Rats of group three (Nano-propolis) received a once-daily stomach gavage of nano-propolis at a rate of 20 mg/kg b.wt. [20] while those of group four (Nano-ginseng) were given a stomach gavage of nano-ginseng at a dose rate of 20 mg/kg b.wt. once daily [21]. Rats of the fifth group (Nano-propolis + dexamethasone) were injected with dexamethasone (7 mg/Kg b.wt. SC) thrice weekly, concurrently with a daily stomach gavage of nano-propolis (20 mg/kg b.wt.). Individual rats of group six (Nano-ginseng + dexamethasone) received the same dose regimen of dexamethasone as those of group two but were gavaged daily with nano-ginseng at a dose of 20 mg/kg b.wt. In the last two groups, individual rats received the same dose regimen of group two concurrently with a once-daily stomach gavage of propolis (20 mg/kg b.wt.) in group seven (Propolis + dexamethasone) or a once daily stomach gavage of ginseng (20 mg/kg b.wt.) in group eight (Ginseng + dexamethasone).

### Bodyweight and gonado-somatic index

Individual rats in all groups were weighed at the end of the experiment. Then, rats were euthanized and testicles were dissected carefully and were trimmed of surrounding tissues. Testes were weighed and were used to calculate the gonado-somatic index according to Foss et al. [22].

### Sampling

Blood samples were obtained by bleeding at the medial canthus of the eye just before euthanasia. Serum was obtained by centrifugation of blood samples at 3500 rpm for 15 min after 12 h. period of refrigerator incubation. Serum samples were kept frozen at − 20 °C till analyses (serum testosterone levels, MDA and TAC). After euthanasia, the following samples were obtained aseptically. Testes and seminal vesicles were obtained for histopathological work, gene analyses and calculation of the gonado-somatic index. The tail of the epididymis was obtained for the estimation of semen parameters.

### Biochemical assays

Serum testosterone concentrations were estimated according to Sato et al. [23] using the radioimmunoassay method. Serum samples were assayed for TAC using commercial kits according to the method described by Koracevic et al. [22] and for MDA levels according to Ohkawa et al. [24]. To do that, particular colorimetric test kits were utilized, following the manufacturer’s instructions (Biodiagnostic Company, Egypt; catalog numbers: MD 25 29 and TA 25 13 for MDA and TAC, respectively). MDA test based on thiobarbituric acid (TBA) reaction in an acidic solution at 95 °C for 30 min. A reactive product of thiobarbituric acid was formed as a result of this reaction. At 534 nm, the absorbance of the resultant colored substance was measured. Concerning TAC, the sample’s antioxidant content is reacted with a predetermined volume of exogenously supplied hydrogen peroxide (H_2_O_2_) to measure TAC. A portion of the H_2_O_2_ was removed from the sample by the antioxidants; the remaining H_2_O_2_ was then colorimetrically detected at 505 nm using an enzymatic process that produced a colored material from 3, 5, dichloro-2-hydroxybenzensulphonate.

### Semen analysis

Semen analysis was carried out according to Aziz et al. [3]. Epididymal semen samples were collected and evaluated for individual sperm motility percentages and sperm concentration. In addition, sperm abnormalities were estimated using the standard eosin-nigrosine staining procedures.

### The quantitative real-time PCR

Total RNA of testicular and seminal vesicle tissue was isolated using EasyRNA™ Cell/Tissue RNA Mini Kit ( Biovision #K1337). The synthesis of first-strand cDNA was done using SuperScript Reverse Transcriptases ( Thermo Scientific ) according to the manufacturer’s instructions. Quantitative PCR was performed using PowerTrack™ SYBR Green Master Mix Applied Biosystems™ on an ABI Prism StepOnePlus Real-Time PCR System (Applied Biosystems) according to the manufacturer’s instructions. The primer sets of the assessed genes were collected in Table 1. The target mRNA expression was normalized to *ACTB* [25].

**Table 1:** Primer sets of assessed genes in the current study

	Sense	Antisense	Ampli-con	Accession no
*StAR*	TGGCTGCCAAAGACCATCAT	TGGTGGGCAGTCCTTAACAC	124	NM_031558.3
*Aromatase*	TGACGTCACTGACAACTCGG	CAAGTCCACGACAGGCTGAT	589	NM_017085.2
*Nrf-2*	TGTAGATGACCATGAGTCGC	TCCTGCCAAACTTGCTCCAT	159	NM_031789.2
*CYP11A1*	GCAAAAGGTCTTTGCCTGCG	TGGATTCTGTGTGTGCCGTT	212	NM_017286.3
*Hsd-3b*	CTCACATGTCCTACCCAGGC	TATTTTTGAGGGCCGCAAGT	362	NM_001007719.3
*ACTB*	CCGCGAGTACAACCTTCTTG	CAGTTGGTGACAATGCCGTG	297	NM_031144.3

### DNA fragmentation

Apoptosis is characterized by fragmentation of chromosomal DNA into multiples of the 180–200 bp nucleosomal unit, known as DNA laddering. The isolated DNA is separated by electrophoresis and visualized using ethidium bromide.

### DNA laddering assay

DNA fragmentation assay was employed as follows: A (200 mg) of the tissue was mechanically homogenized in a hypotonic lysis buffer. The tissue homogenates were then centrifuged, and the pellets comprised total intact DNA (P) while the fragmented DNA was in the supernatants (S). Both fractions were treated separately with 0.5 mL of 25% trichloroacetic acid (TCA) and incubated overnight at 4 °C, then the DNA was assembled by centrifugation. The 80 ul of TCA (5%) is added to each sample and incubated at 90 °C for 20 min. Then, 1 mL diphenylamine reagent was added to each sample and the tubes were incubated overnight at room temperature. The OD was measured at 600 nm. The DNA laddering percentage was calculated using the equation: % DNA fragmentation = [S/(S + P)] × 100 [26].

### Agarose gel electrophoresis of genomic DNA

The agarose gel electrophoresis was done for genomic DNA extracted using the DNeasy kit (Qiagen). The extracted DNA was electrophoresed in 2% agarose gel containing ethidium bromide [27].

### Histopathological examination

By the end of the experiment all animals were sacrificed, the testes and vesicular glands were dissected, washed in normal saline then immersed in Bouin’s solution. The samples were fixed for 24 h. and then transformed for paraffin embedding technique. The first step in this technique was dehydration using ascending graded concentrations of ethanol and then cleared in xylene, impregnated in soft paraffin then embedded in hard paraffin and blocking. By using rotatory microtome the paraffin blocks were sectioned at 2–5 μm thickness. These sections were mounted on clean and dry glass slides then stained with hematoxylin and eosin (H & E) stain. Finally, these stained sections were examined using LEICA (DFC290 HD system digital camera, Heerbrugg, Switzerland) connected to the light microscope using 10 × and 20 × objective lenses [28].

### Statistical analyses

Statistical analysis was performed by SPSS, version 18.0. software, Inc., Chicago, IL, USA. Results were reported as means ± standard errors of the mean (SEM). Means of control and test groups were compared via one-way ANOVA, and post-hoc comparisons were determined using Tukey’s test. The significance level (*P*-value) was set at the 0.05 level.

## Results

### Characteristics of propolis and ginseng and their nano preparation

#### Characteristics of propolis and ginseng and their nano preparation using FT-IR

We used FT-IR as a simple technique to investigate the chemical criteria of propolis and nano-propolis samples (Supplementary Fig. 1A). According to the resulting spectra, there is no significant difference between the spectra of propolis and nano-propolis indicating that the method used to prepare nano-propolis can preserve all the functional groups of propolis. The broad peak that exists at around 3388 cm^−1^ is corresponding to phenolic hydroxyl groups of phenolic compounds or their esters [29, 30]. The doublet peak at 2846/2927 cm^−1^ represents a fingerprint of propolis that reflects the stretching vibrations of C–H bonds in CH3 and CH2 groups [31, 32]. The FT-IR spectra also show a peak at 1630 cm^−1^ resulting from the aromatic ring deformations [29, 32, 33]. The peak at around 1450 cm^−1^ is attributed to the aromatic stretching and C–H deformations of flavonoids [29, 32–34]. Another peak for flavonoids appears at 1050 cm^−1^ which corresponds to C–O bond of aromatic ether [29].

As per the chemical composition of red ginseng and its nanoform, based on the resulting spectra, the spectrum of nano-ginseng sample had the same characteristic peaks of ginseng indicating that the method used to prepare nano-ginseng can preserve all the functional groups of ginseng. The broad peak that exists at around 3400 cm ^−1^ corresponds to the stretching vibration of O–H groups of polysaccharides [35, 36]. The stretching vibration of C–H bond in CH_2_ or CH_3_ (at 2925 cm^−1^), as well as the bending vibration of the same bond (at 1440 cm^−1^), represent the typical structure of a carbohydrate skeleton [35–37]. The peak around 1635 cm^−1^ may be due to the stretching vibration of C=O or C=C also may be attributed to the ionized and esterified carboxyl groups of galacturonic acid [35, 37]. The peak around 1058 cm^−1^ is corresponding to the stretching vibration of C–O and C–H bonds in the guaiacyl unit of lignin [35, 37]. The stretching peak around 1058 cm^−1^ in nano-ginseng sample increased when compared with ginseng, indicating that in nano-ginseng sample, the polymer structures of lignin were degraded [37]. And finally, the broad peak around 576 cm^−1^ represents a fingerprint of carbohydrates [36].

#### XRD patterns of propolis and ginseng and their nano preparation

**Fig. 1:**
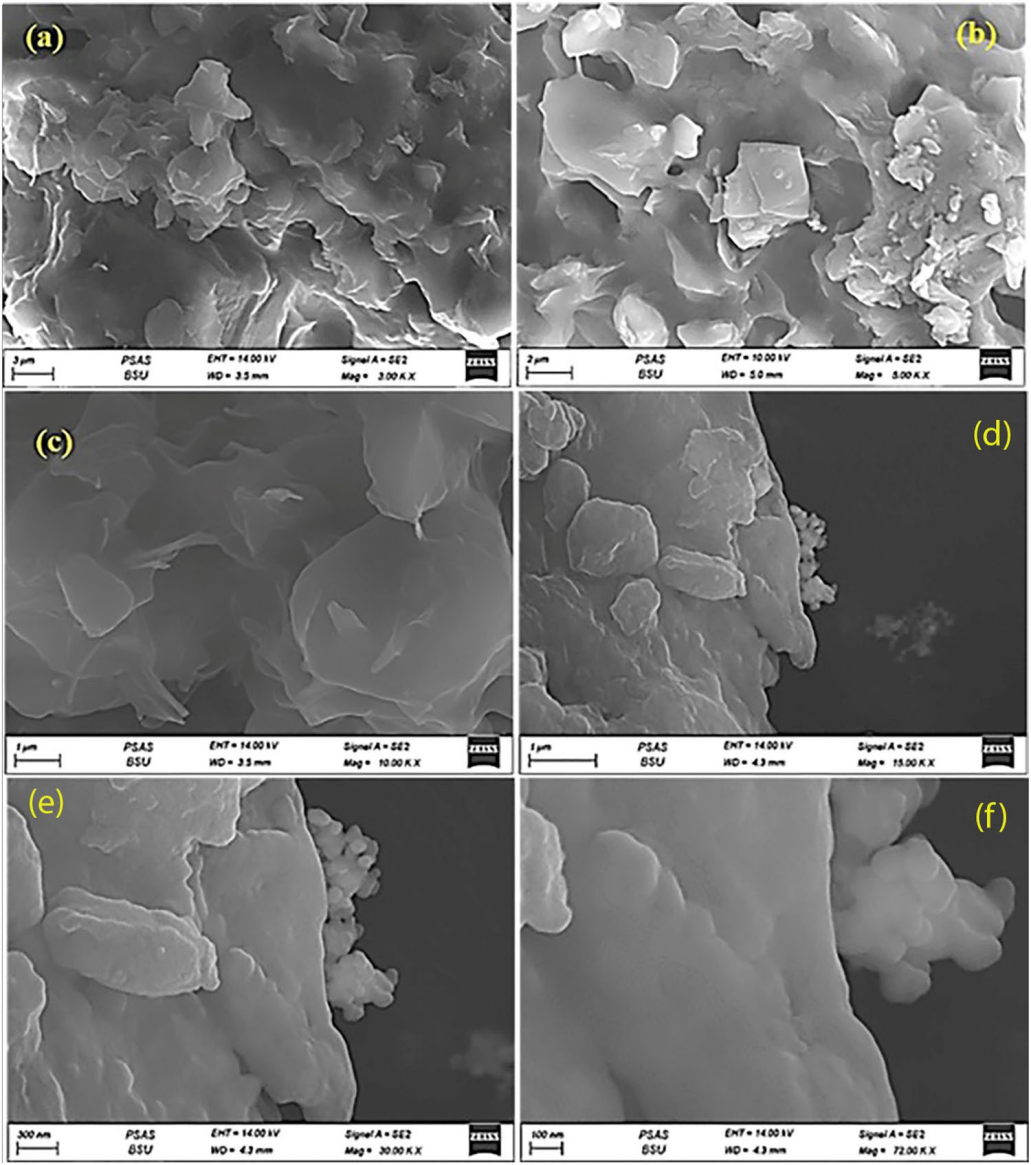
Scanning electron microscpy image of propolis in conventional (**a**–**c**) and nanoparticles (**d**–**f**)

The XRD patterns of propolis, ginseng and their nano are presented in Supplementry Fig. 1B. As reported in previous studies [38–40], the results reflect the amorphous structure of propolis and nano-propolis samples with a broad peak at 2θ ≈ 22°.

Regarding ginseng and nano-ginseng, the results reflect the crystal structure of cellulose in ginseng and nano ginseng samples [35, 36]. In the ginseng sample, the diffraction peaks at 2θ = 12.35°, 13.74° and 19.21° reveal the presence of a parallel chain structure of cellulose type I [41]. On the other hand, the diffraction peaks at 2θ = 13.49°, 19.18° and 21.18° reflect the anti-parallel chain structure of cellulose type II in nano ginseng sample [41–43]. This result indicates that the ball milling process can transform the parallel chain structure of cellulose type I into an anti-parallel chain structure of cellulose type II. Moreover, after converting ginseng into nanoparticles the crystallite size is decreased from 40.3 to 23.6 nm.

#### Scanning electron microscopy of propolis, ginseng and their nano preparation

**Fig. 2:**
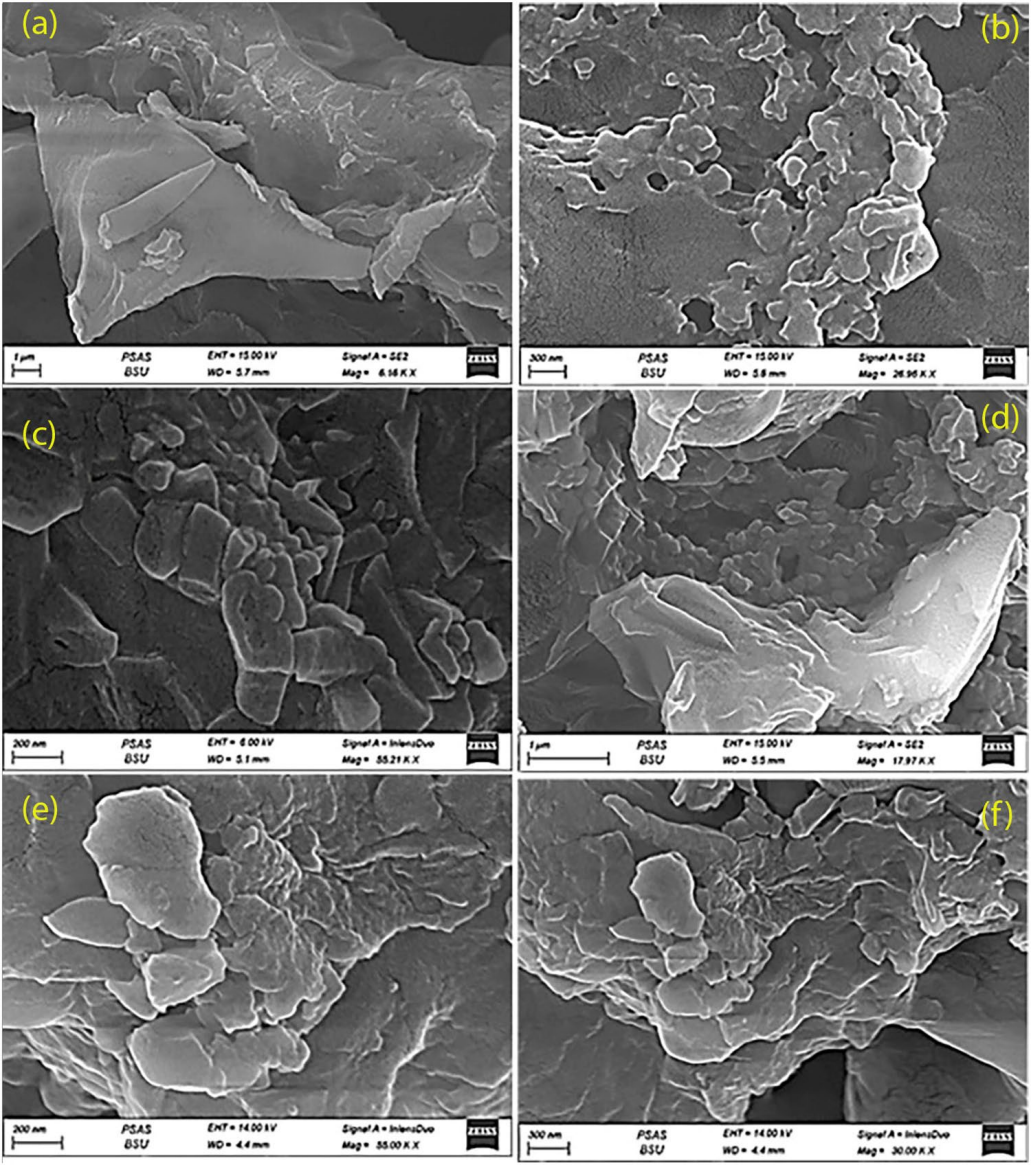
Scanning electron microscpy images of red ginseng in conventional form (**a**–**c**) and nanoparticles (**d**–**f**)

The morphology of the synthesized materials was studied via scanning electron microscopy as demonstrated in Figs. 1 and 2. The layer and sheet propolis are shown in Fig. 1a–c. The images of both propolis and nano-propolis were layered structures, however in the case of ginseng and nano-ginseng present well-defined porous and layered shapes. It is believed that the change in material morphology can be attributed to the different nature and the physical modification via the mailing process.

### Effects of propolis and ginseng either in powder form or as nano-formulations against dexamethasone on the average testes weights, final body weight and gonado-somatic index in adult male albino rats

The results of the average testes weights, final body weight and gonado-somatic index were expressed in Table 2. The mean values of the average testes weights were observed to be comparable among all groups. The final body weights were significantly reduced with dexamethasone treatment in comparison to the control group (*p < 0.05*). On the other side, co-administration of either nano-propolis, nano ginseng or ginseng with dexamethasone achieved significant improvement in the final body weights when compared to the dexamethasone group (*p < 0.05*). Furthermore, the gonado-somatic index exhibited a significant increment with dexamethasone treatment in comparison to the control group (*p* < 0.05).

**Table 2:** Effects of propolis and red ginseng either in powder form or as nano-formulations against dexamethasone on the average testes weights, final body weight and gonado-somatic index in adult male Albino rats (Mean ± SE)

Group	Average testis weight (g)	Final body weight (g)	Gonado-somatic index (GSI)
CTRL –ve	1.34 ± 0.051	272.00 ± 16.85	0.50 ± 0.025
CTRL +ve (dexamethasone)	1.14 ± 0.042	176.00 ± 4.00^a^	0.65 ± 0.20^a^
Nano propolis only	1.33 ± 0.040	264.00 ± 9.27^b^	0.51 ± 0.025^b^
Nano ginseng only	1.26 ± 0.033	248.00 ± 9.17^b^	0.51 ± 0.013^b^
Nano propolis + dexamethasone	1.15 ± 0.085	218.00 ± 8.00^a, b, c^	0.49 ± 0.049^b^
Nano ginseng + dexamethasone	1.16 ± 0.033	230.00 ± 7.75^a, b^	0.50 ± 0.020^b^
Propolis + dexamethasone	1.19 ± 0.047	196.00 ± 4.00^a, c, d^	0.61 ± 0.031
Ginseng + dexamethasone	1.16 ± 0.087	218.00 ± 4.90^a, b, c^	0.54 ± 0.045

The number of animals = 5 for each group

Using one-way ANOVA followed by Tukey post-hoc test

*SE* standard error

^a^*p* < 0.05 versus normal control group

^b^*p* < 0.05 versus dexamethasone group

^c^*p* < 0.01 versus nano propolis only group

^d^*p* < 0.01 versus or nano ginseng only group

### Effects of propolis and ginseng either in powder form or as nano-formulations against dexamethasone on the serum testosterone levels in adult male albino rats

As shown in Table 3, serum testosterone levels were significantly reduced with dexamethasone treatment (*p < 0.01*). Also, it was decreased when the rats were co-administered dexamethasone with either nano-ginseng or propolis (*p < 0.01*). Treating rats with either nano-propolis only or nano-ginseng only induced a significant increase of serum testosterone levels in comparison to both control and dexamethasone groups (*p < 0.01*). Interestingly, co-administration of either nano-propolis or ginseng with dexamethasone succeeded markedly in modulating the serum testosterone levels that were significantly altered with dexamethasone (*p < 0.01*).

**Table 3:** Effects of propolis and red ginseng either in powder form or as nano-formulations against dexamethasone on serum testosterone level, semen quality and oxidative markers (Mean ± SE)

Group	Testosterone (ng/ml)	Individual motility (%)	Sperm concentration ( ×10^6^/ml)	Sperm abnormalities (%)	MDA nmol/ml	(TAC) mM/L
CTRL –ve	2.62 ± 0.061	84.60 ± 1.72	14.20 ± 0.86	10.00 ± 0.71	1.12 ± 0.07	1.34 ± 0.035
CTRL +ve (dexamethasone)	1.05 ± 0.029^a^	55.60 ± 1.36^a^	4.40 ± 0.51^a^	30.80 ± 1.28^a^	1.88 ± 0.08^a^	0.79 ± 0.044^a^
Nano propolis only	2.98 ± 0.030^a, b^	87.80 ± 1.50^b^	13.20 ± 0. 37^b^	8.20 ± 0. 86^b^	0.79 ± 0.04^a, b^	1.63 ± 0.040^a, b^
Nano ginseng only	3.01 ± 0.033^a, b^	86.4 ± 1.63^b^	14.00 ± 0.71^b^	10.60 ± 0.75^b^	0.77 ± 0.05^a, b^	1.67 ± 0.052^a, b^
Nano propolis + Dexamethasone	2.88 ± 0.027^a, b^	83.80 ± 1.91^b^	13.60 0.68^b^	12.60 ± 0. 87^b^	0.90 ± 0.04^b^	1.62 ± 0.046^a, b^
Nano ginseng + Dexamethasone	1.02 ± 0.032^a, c, d^	57.20 ± 1.77^a, c, d^	8.6 ± 0.51^a, b, c, d^	25.20 1.16^a, b, c, d^	1.89 ± 0.10^a, c, d^	0.87 ± 0.057^a, c, d^
Propolis + Dexamethasone	1.03 ± 0.016^a, c, d^	57.20 ± 1.77 ^a c d^	7.80 ± 0.73^a, b, c, d^	25.60 ± 1.20^a, b, c, d^	1.96 ± 0.06^a, c, d^	0.79 ± 0015^a, c, d^
Ginseng + Dexamethasone	2.80 ± 0.022^a, b, c, e^	86.00 ± 2.61^b, e^	12.80 ± 0.58^b, e^	13.20 ± 0.58^b, e^	1.07 ± 0.02^b, d, e^	1.38 ± 0.012^b, c, d, e^

The number of animals = 5 for each group

Using one-way ANOVA followed by Tukey post-hoc test

*SE* standard error, *MDA* malondialdehyde, *TAC* total antioxidant capacity

^a^*p* < 0.01 versus normal control group

^b^*p* < 0.01 versus dexamethasone group

^c^*p* < 0.05 versus either nano proplis only or nano ginseng only group

^d^*p* < 0.05 versus nano proplis + dexamethasone group

^e^*p* < 0.01 versus either nano ginseng + dexamethasone group or proplis + dexamethasone groups

### Effects of propolis and ginseng either in powder form or as nano-formulations against dexamethasone on the serum concentrations of malondialdehyde and total antioxidant capacity in adult male albino rats

The findings in Table 3 illustrated that dexamethasone treatment exhibited a significant elevation of the serum levels of MDA and significant reduction of those of TAC concerning control group (*p < 0.01*). On the contrary, administration of nano-propolis only and nano ginseng only improved noticeably the oxidative status as they induced significant increases in serum levels of TAC and decreased those of MDA in comparison to both control and dexamethasone groups (*p < 0.01*). The most striking finding was that nano-propolis and ginseng when co-administered with dexamethasone, achieved an interesting ameliorative effect on the oxidative stress induced with dexamethasone via enhancing the serum levels of TAC and declining those of MDA (*p < 0.01*).

### Effects of propolis and ginseng either in powder form or as nano-formulations against dexamethasone on semen quality in adult male albino rats

Based on information illustrated in Table 3 and Supplementary Fig. 5, dexamethasone treatment impaired significantly the semen quality as it reduced noticeably the percentage of individual motility and sperm cells concentration along with elevation of the percentage of sperm cell abnormalities (*p < 0.01*). Amazingly, nano-propolis and ginseng co-administration with dexamethasone alleviated significantly the impairment effects of dexamethasone on sperm cells. They induced a significant elevation of the percentage of individual motility and sperm cell concentration along with significant reduction of sperm cell abnormalities when compared with the dexamethasone group (*p < 0.01*).

### Effects of propolis and ginseng either in powder form or as nano-formulations against dexamethasone on the mRNA expression levels of *CYP11A1*, *ARO*, *HSD-3b*, *StAR*, and *Nrf-2* genes in the testes and seminal vesicles

**Fig. 3:**
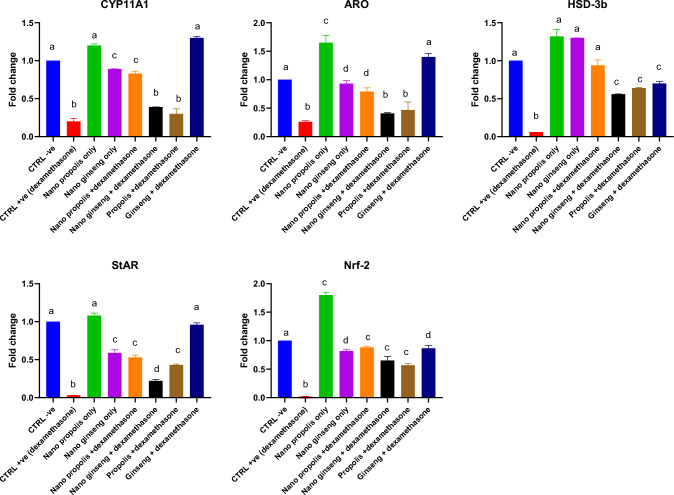
Effects of nano-particles of propolis and red ginseng either alone or in combination with dexamethasone on the testicular mRNA expression levels of *CYP11A1*, *ARO*,* HSD-3b*, *StAR* and *Nrf-2* (Each bar represents mean ± SE)

As shown in Fig. 3, dexamethasone treatment resulted in a marked downregulation of the testicular mRNA expression levels of *CYP11A1*, *ARO*, *HSD-3b*, *StAR* and *Nrf-2*. It has been noticed that all treatments other than nano-ginseng + dexamethasone and propolis + dexamethasone counteracted the negative effects of dexamethasone and upregulated significantly the testicular mRNA expression levels of *CYP11A1* in comparison to the dexamethasone group (*p < 0.05*). Concerning *HSD-3b*, *StAR*, *ARO* and *Nrf-2*, their testicular mRNA expression levels were significantly upregulated by all treatments in comparison to the dexamethasone group. However, the nano-propolis + dexamethasone treatment achieved better results in neutralizing the negative effects of dexamethasone than propolis + dexamethasone while ginseng + dexamethasone attained superior effects in comparison to nano-ginseng + dexamethasone.

The gene expression analyses in seminal vesicles were illustrated in Supplementary Fig. 6. The mRNA expression levels of *CYP11A1*, *ARO*, *HSD-3b*, *StAR* and *Nrf-2* showed significant decrement with dexamethasone treatment. It has been noticed that all treatments except for propolis + dexamethasone succeeded significantly in abolishing the downregulating effects of dexamethasone on the mRNA expression levels of *CYP11A1*, *HSD-3b* and *StAR*. However, the best results were attained with either nano-propolis only, nano ginseng only ,or ginseng + dexamethasone treatments. Regarding the mRNA expression levels of *ARO*, it was found that nano-propolis only, nano-ginseng only and ginseng + dexamethasone reversed the negative effects of dexamethasone remarkably and recovered the levels to control ones (*p < 0.05*). Although all treatments ameliorated the effects of dexamethasone on the mRNA expression levels of *Nrf-2*, nano-propolis only, nano-ginseng only and ginseng + dexamethasone were the best ones as they upregulated the *Nrf-2* levels to be comparable with the control values.

### Effects of propolis and ginseng either in powder form or as nano-formulations against dexamethasone on the DNA fragmentation percentages in testicular and semen vesicles glandular tissues in adult male albino rats

**Fig. 4:**
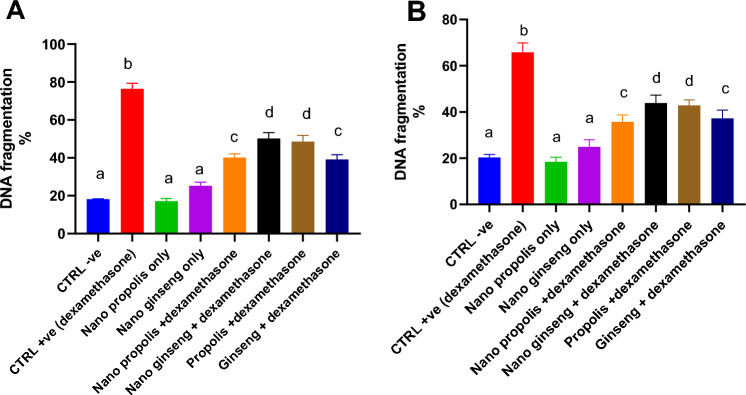
Effects of nano-particles of propolis and red ginseng either alone or in combination with dexamethasone on the DNA fragmentation percentages in testicular (**A**) and semen vesicles glandular (**B**) homogenates (Each bar represents mean ± SE). *SE* Standard error. Different superscripts within parameter were different (*p* <0.05). The DNA fragmentation assay was performed using DPA (diphenylamine). DNA fragmentation percentages were calculated (wavelength 590 nm)

As demonstrated in Fig. 4 and Supplementary Fig. 7, dexamethasone administration increased significantly the percentages of DNA fragmentation in both testicular and semen vesicle glandular tissues (*p < 0.05*). On the other side, all treatments improved significantly these percentages in comparison to the dexamethasone group (*p < 0.05*). Nano propolis only and nano ginseng only were potent enough to keep the percentages of DNA fragmentation comparable to control levels. Both nano-propolis + dexamethasone and ginseng + dexamethasone treatments accomplished more powerful effects against dexamethasone when compared to propolis + dexamethasone and nano-ginseng + dexamethasone.

### Effects of propolis and ginseng either in powder form or as nano-formulations against dexamethasone on the histopathological findings in both testes and seminal vesicles

**Fig. 5:**
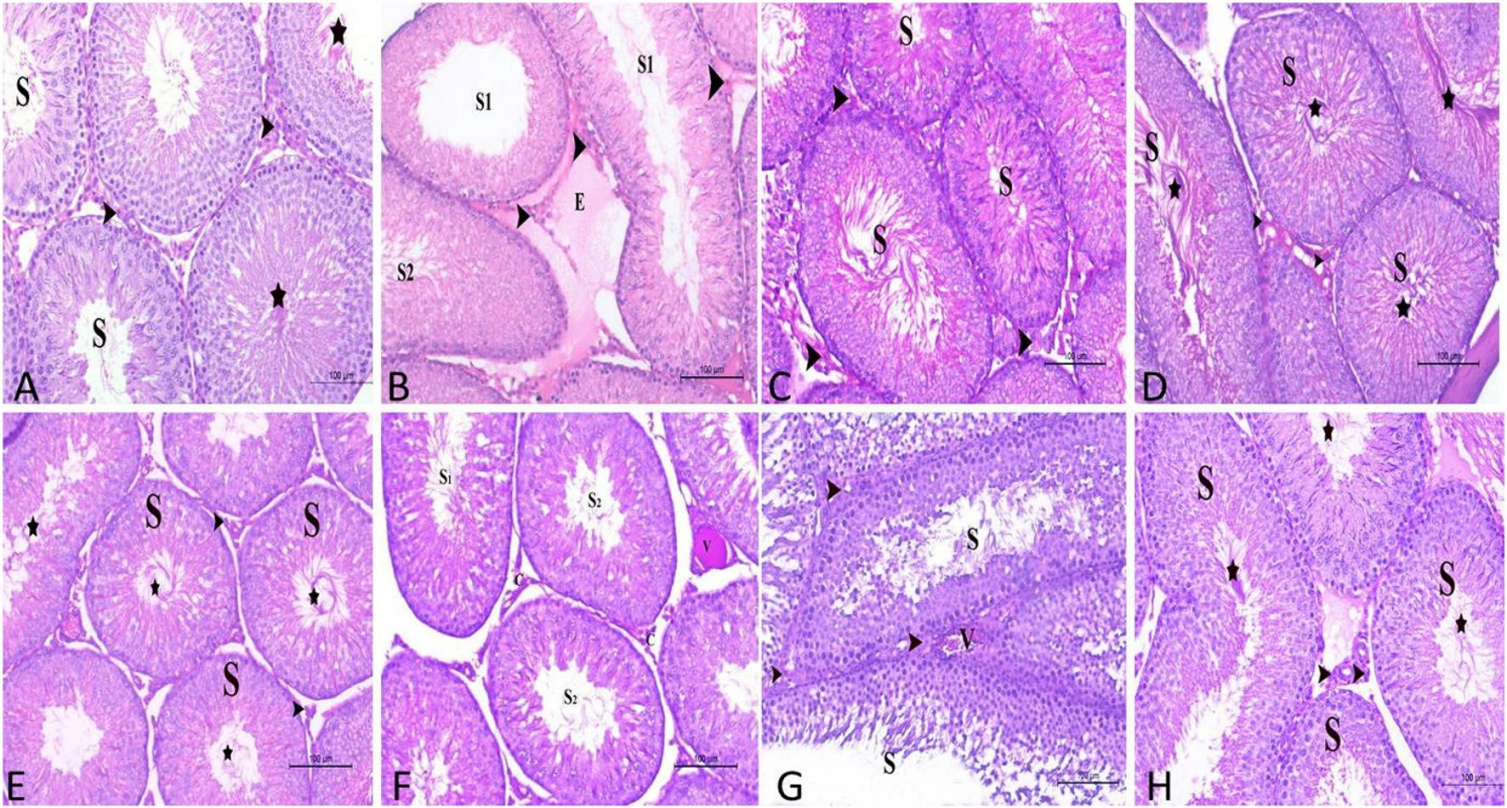
A histological section in the testis of adult male albino rats stained with H&E X200. **A** Control group showed normal seminiferous tubules (S) containing normal spermatogenic cells and huge amount of spermatid (star). The normal interstitial tissue contained blood capillaries and active Leydig cells (arrow head). **B** Dexamethasone- treated group showed normal seminiferous tubules (S1) and other seminiferous tubules (S2) lined with spermatogenic cells suffered from different degrees of degenerative changes, few amounts of spermatid and sperms. Note, the interstitial tissue showed a marked degree of edema (E) and inactive Leydig cells (arrow head). **C** Nano-propolis only group showed normal seminiferous tubules (S) that were lined with normal highly proliferative spermatogenic cells and huge amount of spermatid and sperms. The normal interstitial tissue contained blood capillaries and highly active Leydig cells (arrow head). **D** Nano ginseng only group exhibited normal seminiferous tubules (S) that were lined with normal highly proliferative spermatogenic cells and numerous spermatid and sperms (star). The normal interstitial tissue contained blood capillaries and highly active Leydig cells (arrow head). **E** Nano-propolis +dexamethasone group showed normal seminiferous tubules (S) with normal spermatogenic cells and huge amount of spermatid (star). The normal interstitial tissue contained highly active Leydig cells (arrow head). **F** Nano ginseng + dexamethasone group showed that the majority of seminiferous tubules (S) were lined with degenerated spermatogenic cells, few amounts of spermatid and sperms. The interstitial tissue showed congestion of the testicular blood vessels (V) and inactive Leydig cells (arrow head). **G** Propolis + dexamethasone group showed that the majority of seminiferous tubules (S) lined with spermatogenic cells with degenerative changes, few amounts of spermatid and sperms. Note, the interstitial tissue showed marked degree of edema, congestion of blood vessels (V) and the Leydig cells appeared less active (arrow head). (**H** and **E**) stain X200. **H** Ginseng + dexamethasone group displayed normal seminiferous tubules (S) containing normal spermatogenic cells and huge amount of spermatid and sperms (star). The interstitial tissue contained less congested blood capillaries and active Leydig cells (arrow head)

 Administration of dexamethasone remarkably altered the histological appearance of the testicular tissue and semen vesicular gland. The testicular seminiferous tubules appeared to suffer from different degrees of degenerative changes in the spermatogenic cells resulting in few amounts of spermatids and sperms appearing in the lumen of these tubules. The interstitial tissue showed a marked degree of edema and congestion of the blood vessels. The Leydig cells showed prominent degrees of inactivity (Fig. 5B). The use of ginseng and nano-propolis ameliorated the action of dexamethasone on testicular and vesicular gland structure by increasing the proliferation of spermatogenic cells resulting in the observation of huge amounts of spermatids and sperms in the tubular lumen. The interstitial tissue contained normal blood capillaries and numerous highly active Leydig cells (Fig. 5E and H). On contrary, using of nano ginseng and propolis did not counteract the negative effects of dexamethasone on testicular tissue as a high degree of degeneration in spermatogenic cells, few amounts of sperms and reduced activity of Leydig cells were excessively noticed (Fig. 5F and G). The same results were observed in the vesicular gland structure. The gland acini in the dexamethasone group appeared small, collapsed and inactive. The acini are lined with low columnar epithelium with few secretory activities. The interstitial connective tissue became highly proliferative and abundant around the acini (Supplementary Fig. 8B). Administration of ginseng and nano propolis ameliorated the action of dexamethasone by decreasing the proliferation of connective tissue between the gland acini, increasing the size of these acini as well as increasing the secretory activity of acinar epithelia resulting in the accumulation of secretory materials on the acinar lumen (Supplementary Fig. 8E and H). On the contrary, using of nano ginseng and propolis failed to ameliorate the effects of dexamethasone as they increased the proliferation of the interstitial connective tissue around the acini which appeared small and collapsed with few secretory materials and lined with less active secretory cells (Supplementry Fig. 8F and G).

## Discussion

We designed this study to determine if nano-preparations from two natural compounds known for their antioxidant properties, propolis and red ginseng, would be more effective than their conventional forms in ameliorating the oxidative stress induced by dexamethasone in the male reproductive system of intact mature albino rats.

Serum testosterone levels were significantly reduced in response to dexamethasone treatment in the control positive group as shown in Table 3. This finding is supported by findings from previous studies in albino rats [5], mice [9], Wister rats [44], breeder roosters [45] and stallions [10]. Lower levels of testosterone in dexamethasone-treated males might be due to suppressed biosynthesis of testosterone in interstitial cells of Leydig mediated by NR3C1 as cited by Zhang et al. [46]. In that study, the authors found also that glucocorticoids targeted stem Leydig cells inhibiting their differentiation and accordingly reducing the levels of testosterone. In addition, our findings regarding gene analysis could explain the negative effects of dexamethasone on serum testosterone concentration through downregulation of the testicular transcript levels of *CYP11A1*, *HSD-3b* and *StAR* which are necessary for steroidogenesis.

Serum testosterone levels were significantly increased in the two groups treated with nano- propolis only and nano-ginseng only as compared to the control negative group. Similar to these findings, a previous study revealed that serum testosterone levels were higher in rats administered with ginseng nano-particles at dose rates of 10 or 20 mg/kg b.wt, compared to the control negative group, a meanwhile lower dose of ginseng nano-particles (4 mg/kg b.wt.) did not affect serum testosterone levels [21]. Research on the effects of propolis nano-particles on serum testosterone levels is lacking to the best of our knowledge. Ginseng nano-particles might have raised serum testosterone levels due to the possible potent effects of the nano-formulation as the conventional red ginseng has been reported to protect the hypophyseal-pituitary-gonadal axis against various insults provoking stress [47].

Regarding the ameliorative effects against dexamethasone, propolis nano-particles and conventional ginseng were associated with higher serum testosterone levels, when compared to other groups. Conversely, conventional propolis and nano-ginseng did not restore serum testosterone levels to normal values when co-administered with dexamethasone. The fact that propolis nano-particles, but not conventional propolis, improved serum testosterone levels could be explained by our gene analysis findings, which showed that, unlike conventional propolis, propolis nano-particles significantly increased the testicular transcript levels of *CYP11A1*, which were markedly reduced by dexamethasone.

On the other hand, nano-ginseng did not counteract the effects of dexamethasone on testosterone levels. It has been cited that ginsenosides partially reversed the downregulation provoked by dexamethasone on its target intracellular glucocorticoid receptors [48]. So, it is likely that nanoparticles of ginseng have been more able to reverse the downregulation provoked by dexamethasone on glucocorticoid receptors as they had greater opportunities to penetrate intracellularly, thereby potentiating the effects of dexamethasone, rather than counteracting its effects [25]. Furthermore, our findings revealed that nano-ginseng was unable to reverse dexamethasone’s downregulation of *CYP11A1* transcript levels in the testes, which is required for steroidogenesis. However, when the animals were given conventional ginseng with dexamethasone, the significant upregulation of the testicular transcript levels of *CYP11A1* and *StAR* was obvious. This might explain why conventional ginseng, but not nano-ginseng, could reverse the negative effects of dexamethasone on serum testosterone levels.

The seminal parameters studied in the current study were significantly decreased in dexamethasone-treated rats, compared to the control group. Similar negative effects of dexamethasone on semen pictures were observed in previous studies [11, 45]. While, in stallions, two doses of dexamethasone did not significantly alter seminal parameters three days after treatment [10]. The lower seminal parameters observed in the dexamethasone-treated group in our study might be due to the change in the number of testicular germ cells along with alteration of their differentiation [49].

While propolis and nano-ginseng did not protect against dexamethasone-induced changes in semen parameters, nano-propolis and conventional ginseng mitigated dexamethasone’s detrimental effects. Failure of nano-ginseng, not ginseng, to ameliorate the negative effects of dexamethasone on testicular environment was discussed with testosterone concentrations.

Conventional red ginseng showed ameliorating influences against dexamethasone toxicity on semen picture in the current study. Ginsenosides are agonists of the GABA(A) receptor in receptor-ligand binding experiments [50]. This may imply that ginseng can influence the pituitary-testicular axis on hormonal and neuronal levels. Hence, it could be inferred that nanogiensing formulations increased penetration into the cell membrane, it can’t effectively activate the extracellular receptors for acetylcholine and dopamine, which intercalated in the reproductive pattern. Ginseng extracts include a combination of ginsenosides that can activate either or both ER and PR, modulating several aspects of sperm activity [51]. The progesterone receptors integrated into the membrane were found to elicit rapid activation of cellular signaling pathways upon binding of the extracellular ligand, so the ginseng needs to be in conventional form, not the nano form, to act well in these extracellular receptors.

Our finding reported that dexamethasone treatment provoked oxidative stress via elevation of the serum levels of MDA and reduction of those of TAC [52, 53]. The action of dexamethasone to induce oxidative stress could be attributed to its ability to reduce the enzymatic and nonenzymatic antioxidants [5]. In addition, dexamethasone was found to downregulate the mRNA expression levels of *Nrf-2* which is a potent antioxidant factor [54].

On the contrary, administration of nano-propolis only and nano-ginseng only improved noticeably the oxidative status. In this concern, nano propolis was observed to reduce the levels of MDA and elevate the enzymatic antioxidants including catalase and glutathione peroxidase [55]. To the best of our knowledge, there are no previous studies that examined the improving effects of nano ginseng only on oxidative stress. The modulating effects of nano- propolis only and nano-ginseng only on oxidative stress could be explained by our results regarding gene analysis that revealed a marked upper regulation of the mRNA expression levels of *Nrf-2*.

Interestingly, nano-propolis and conventional ginseng but not propolis and nano ginseng significantly ameliorated the oxidative stress-induced with dexamethasone. In this concern, feeding aged rats with a diet containing red ginseng improved noticeably the oxidative stress by elevation of both enzymatic and nonenzymatic antioxidants [56]. Also, red ginseng was effective in counteracting the negative effect of ethanol-inducing oxidative stress in hepatic tissues via activation of AMPK/Sirt1. Additionally, by evaluating our gene analysis data, we could explain the improving effects of nano-propolis and conventional ginseng against dexamethasone-induced oxidative stress by their ability to upregulate the mRNA expression levels of *Nrf-2* that reduced markedly with dexamethasone.

The mean values of the average testes weights were observed to be comparable among all groups. The final body weights were significantly reduced with dexamethasone treatment in comparison to the control group (*p < 0.05*). A study by Hasona et al. [57] showed that dexamethasone reduced significantly the body weight gain of rats. Furthermore, the GSI was increased significantly with dexamethasone in comparison to the control group. This might be due to the marked reduction in body weight by dexamethasone along with constant mean values of average testes weights.

On the other side, co-administration of either nano propolis, nano ginseng, or ginseng with dexamethasone improved significantly the final body weights. ALginate-propolis nanoparticles were observed to improve body weight gain greater than propolis [58]. In addition, ginseng succeeded markedly in switching the deviation in body weight of rabbits caused by mercuric chloride to the normal state [59]. The improving effects of nano propolis, nano ginseng and ginseng on body weight might be due to their antioxidant properties [55].

In this study, some steroidogenesis-related genes were investigated in both seminal vesicles and testes of the rat. *StAR* (Steroidogenic acute regulatory) gene regulates the rate-limiting step in steroid biosynthesis. It regulates the delivery of cholesterol from the outer to the inner mitochondrial membrane, so it plays an important role in the regulation of steroid biosynthesis [60]. *Aromatase* gene is also called estrogen synthase or *CYP19A1* gene. It regulates the rate-limiting step of estrogen biosynthesis. It catalyzes the aromatization of androgens to estrogens [61]. *CYP11A1* (Cytochrome P450 Family 11 Subfamily A Member 1) is a protein-coding gene that catalyzes the conversion of cholesterol to pregnenolone, the precursor of most steroid hormones [62]. *HSD-3b* (Hydroxy-Delta-5-Steroid Dehydrogenase, 3 Beta- And Steroid Delta-Isomerase 1) is a protein-coding gene that catalyzes the oxidative conversion of delta-5-3-beta-hydroxysteroid precursors into delta-4-ketosteroids which lead to the production of all classes of steroid hormones [62, 63]. We also investigated the expression of the *Nrf-2* gene which regulates the GSH synthesis and maintenance which reflect the condition of oxidative stress in the seminal vesicle and testes of rats [64]. The process of steroidogenesis initiates with the conversion of cholesterol to pregnenolone by cholesterol side-chain cleavage enzyme cytochrome P450 (*CYP11A1*) within the mitochondria. StAR facilitates the transport of cholesterol within the mitochondria. Pregnenolone is then catalyzed into other steroids by a series of oxidative enzymes located in both mitochondria and endoplasmic reticulum. The accessibility of these enzymes in a given tissue determines the resultant functional steroids in a given gland or tissue [61, 65]. The transcript levels of *CYP11A1, ARO, HSD-3b, StAR and Nrf-2* were down-regulated in seminal vesicles and testicular tissues by the effect of dexamethasone. This result was favored by Koibuchi et al. [66] who reported the down-regulation of the expression of *CYP11A1, HSD-3b*, and *StAR* genes by the effect of dexamethasone in human glioma GI-1 cells. Dexamethasone inhibits the activation of these genes’ expression through the inhibition of ACTH (Adrenocorticotrophic hormone) which is responsible for steroid output from the adrenal gland to circulation [66]. Dexamethasone counteracts the induced activation of *Nrf-2* target genes in a GR- (glucocorticoid receptor) dependent manner. The antioxidant response of *Nrf-2* is impaired by dexamethasone resulting in a decrease in the cellular antioxidant capacity [67]. The major factor of oxidative stress in the cell is DNA fragmentation [68]. In our study, we examined the DNA fragmentation in both testicular and seminal vesicle glandular tissues of rats which were very clear due to the effect of dexamethasone. This result is counteracted by the result of Baumeister et al. [68] which demonstrated the chemoprotective action of dexamethasone in oxidatively stressed cells. Our results suggested the positive effect of ginseng and nano propolis against dexamethasone in testes while reporting the most powerful effect of ginseng in the seminal vesicle. Nna et al. [68] reported that propolis upregulated the mRNA level of *CYP11A1, HSD-3b*, and *StAR* genes. Propolis suppressed the ROS-associated effects. This may explain the upregulation of *Nrf-2* by propolis. Propolis improved steroidogenesis through the activation of the rate-limiting step and the enzymes that catalyze the subsequent reactions in steroidogenesis [69]. Kim et al. [70] suggested that ginseng improved the testes’ function through *CYP11A1* which regulates the upregulation of other genes; *HSD-3b* and *StAR.* Ginseng stimulated spermatogenesis and activated glial cell-derived neurotrophic factor and cAMP-responsive element modulator in rat testes. Ginseng ameliorated the aging effect on testes by regulating the *CYP11A1* which is related to C21-steroid hormone metabolism [70]. Yangi et al. [71] showed that propolis attenuated the inflammatory responses, oxidative stress, and DNA fragmentation in the rat. Kang et al. [71] investigated the positive effect of red ginseng which appeared in reducing ROS and apoptotic indices (cell death and DNA fragmentation). Seven et al. [55] reported the ameliorative effect of propolis and nano-propolis on oxidative stress and apoptosis in rats with testicular damage. This result confirmed that nano-propolis was more effective than free propolis. Nano propolis has better solubility and chemoprotection than propolis [55] which may be related to the most powerful antioxidant property of nanoparticles. This result agreed with our results about nano propolis which exerts the best effect in the testicular tissue while this result was not achieved in the seminal vesicle. On the other hand, ginseng exerted a better effect than nano ginseng in the seminal vesicle which counteracts the evolution of nanotechnology.

## Conclusion

In conclusion, propolis in nano-form and conventional ginseng could be considered promising treatments for mitigating all of the negative effects of dexamethasone on male reproductive capacity. The most striking discovery was that co-administration of both treatments with dexamethasone significantly switched the expression of *CYP11A1*, *StAR*, *HSD-3b*, *Nrf-2* *ACTB*-3b genes in the testicular and seminal gland tissues from deviation to normal state. However, neither nano-ginseng nor conventional propolis had the same calming effect.

## Supplementary Information

Below is the link to the electronic supplementary material.

Supplementary material 1 (DOCX 1399.8 kb)

## Data Availability

The data that support the findings of this study are available on request from the corresponding. author.
