# Supplementary material for: Physiological roles of propolis and red ginseng nanoplatforms in alleviating dexamethasone-induced male reproductive challenges in a rat model
Source: Mol Biol Rep. 2024 Jan 4;51(1):72. doi: 10.1007/s11033-023-08991-4 (PMC10766727; doi:10.1007/s11033-023-08991-4)
Supplement: Supplementary file 1 — Supplementary material 1 (DOCX 1399.8 kb) [file 11033_2023_8991_MOESM1_ESM.docx]

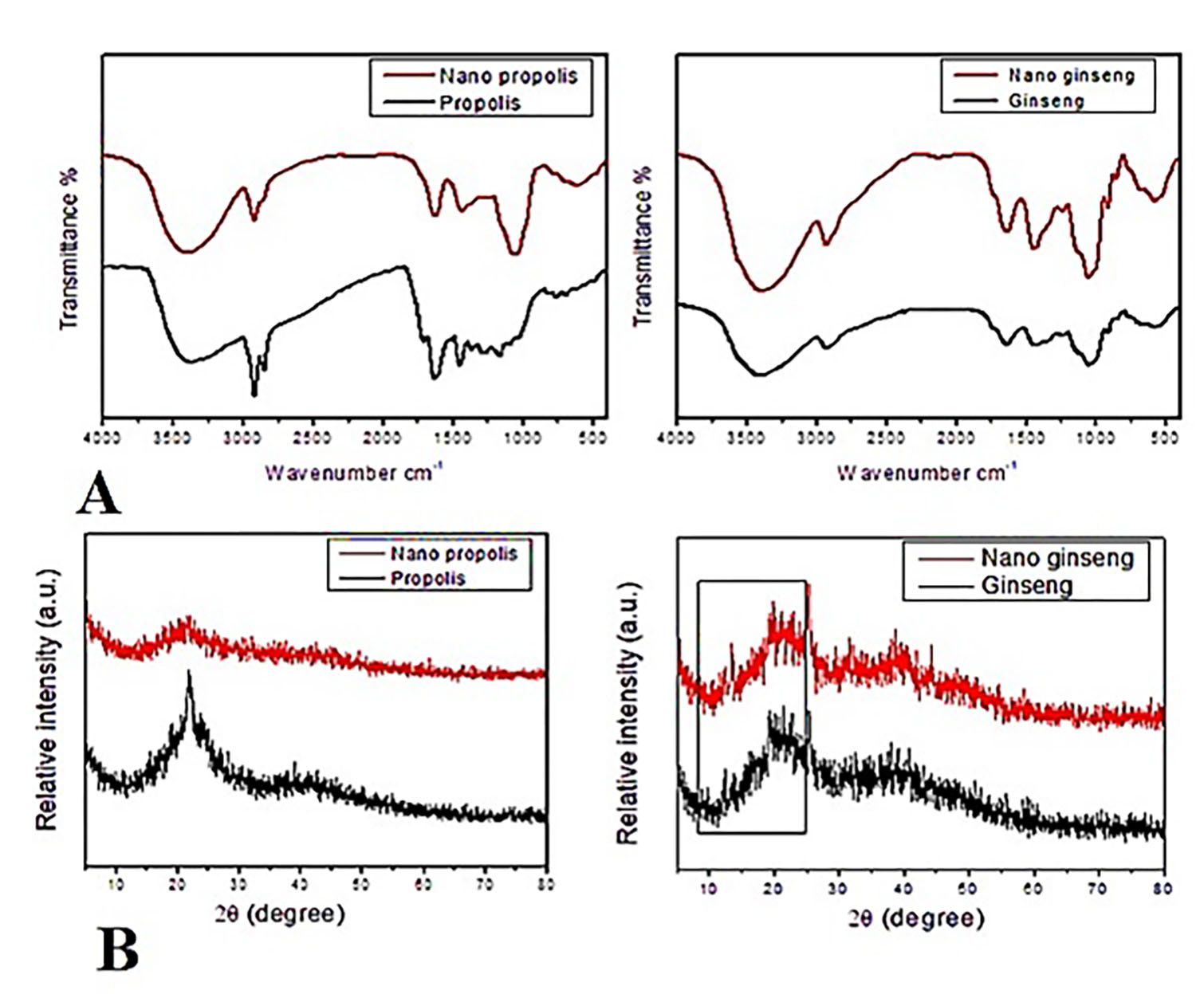


**Supplementary Figure 1.** FTIR spectra of propolis and red ginseng in conventional and nanoplatforms (A) and XRD patterns of propolis and red ginseng in conventional and nanoplatforms (B).


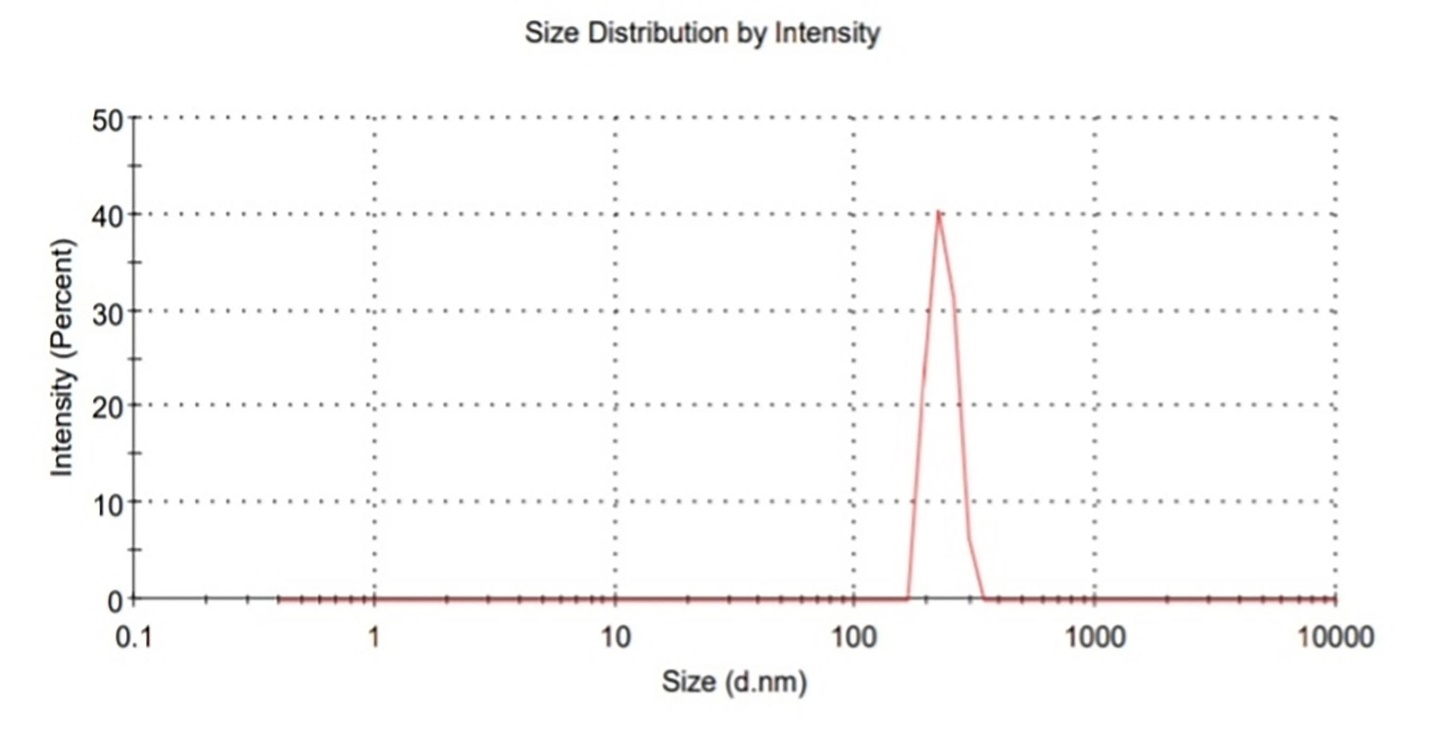


**Supplementary Figure 2.** Zeta size distribution of nano-propolis.


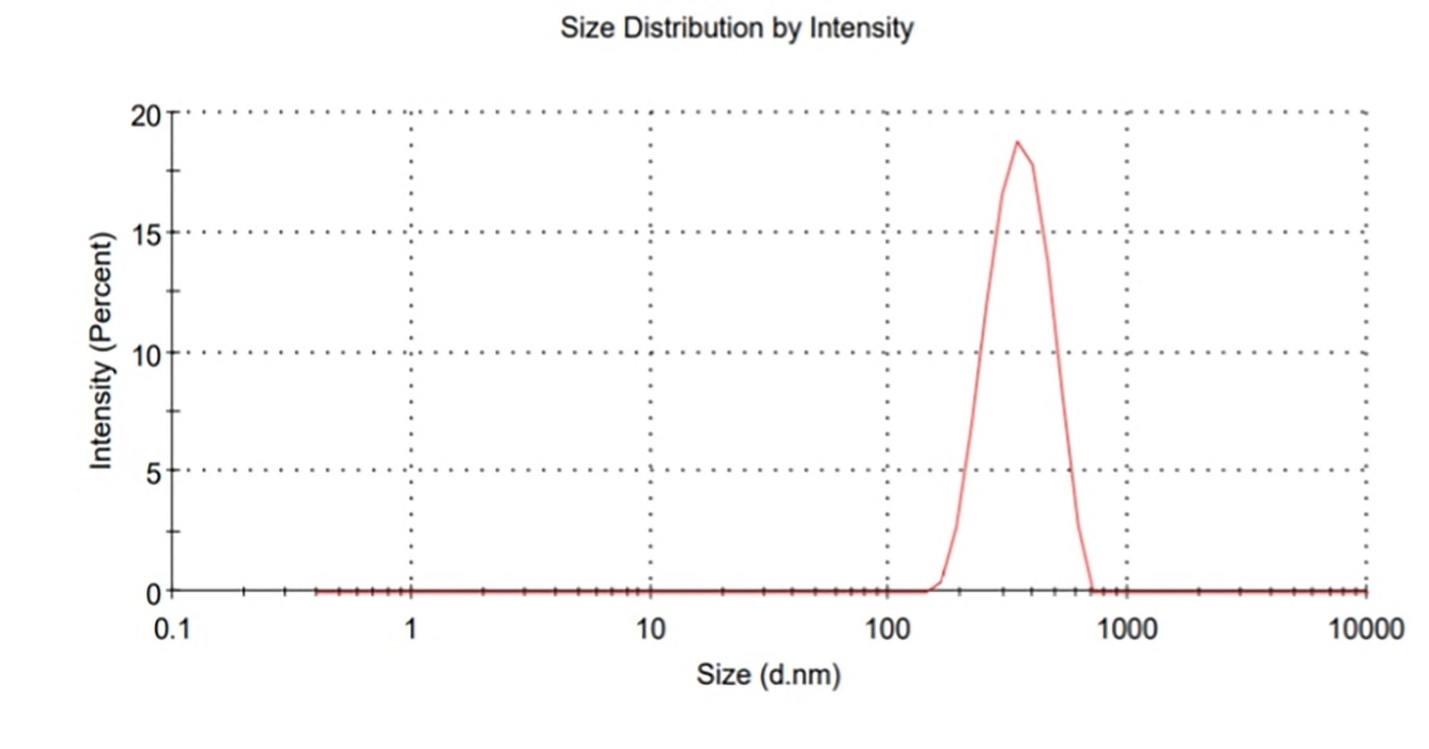


**Supplementary Figure 3.** Zeta size distribution of nano-ginseng.


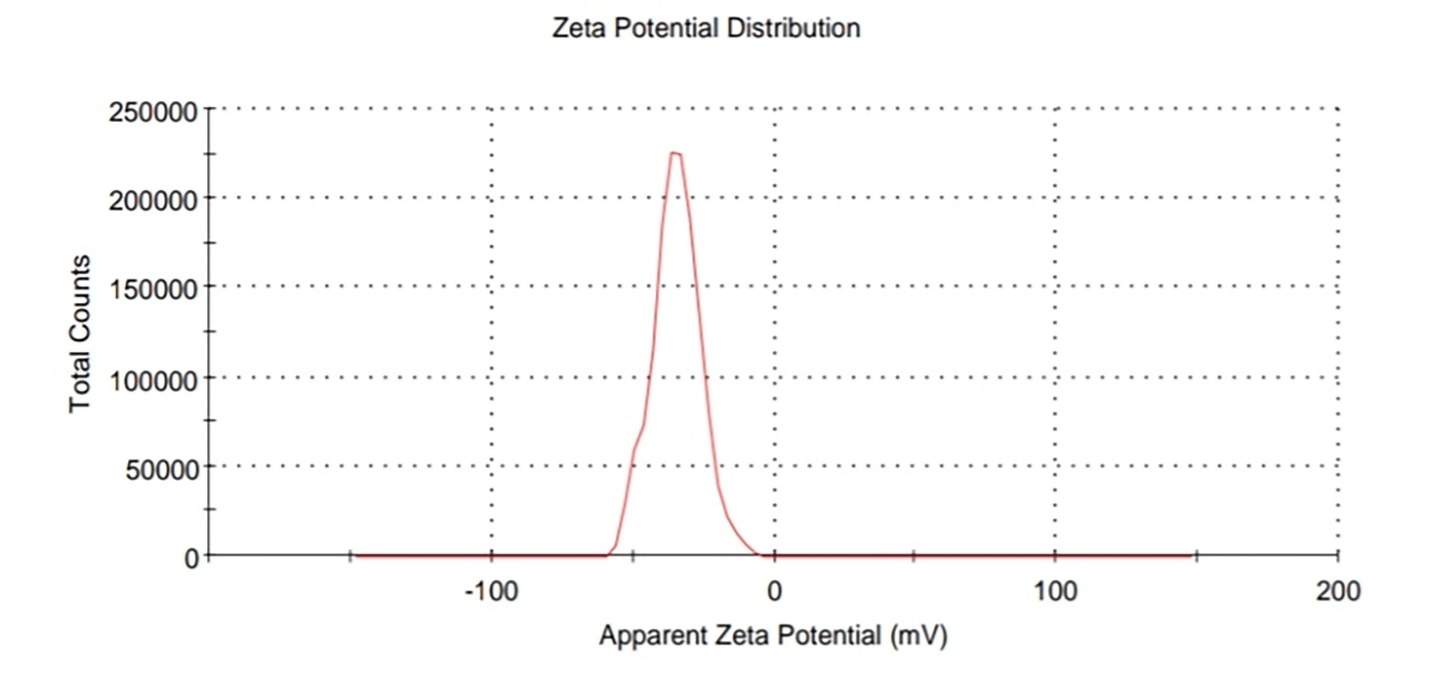


**Supplementary Figure 4.** Zeta potenial of nano-propolis.


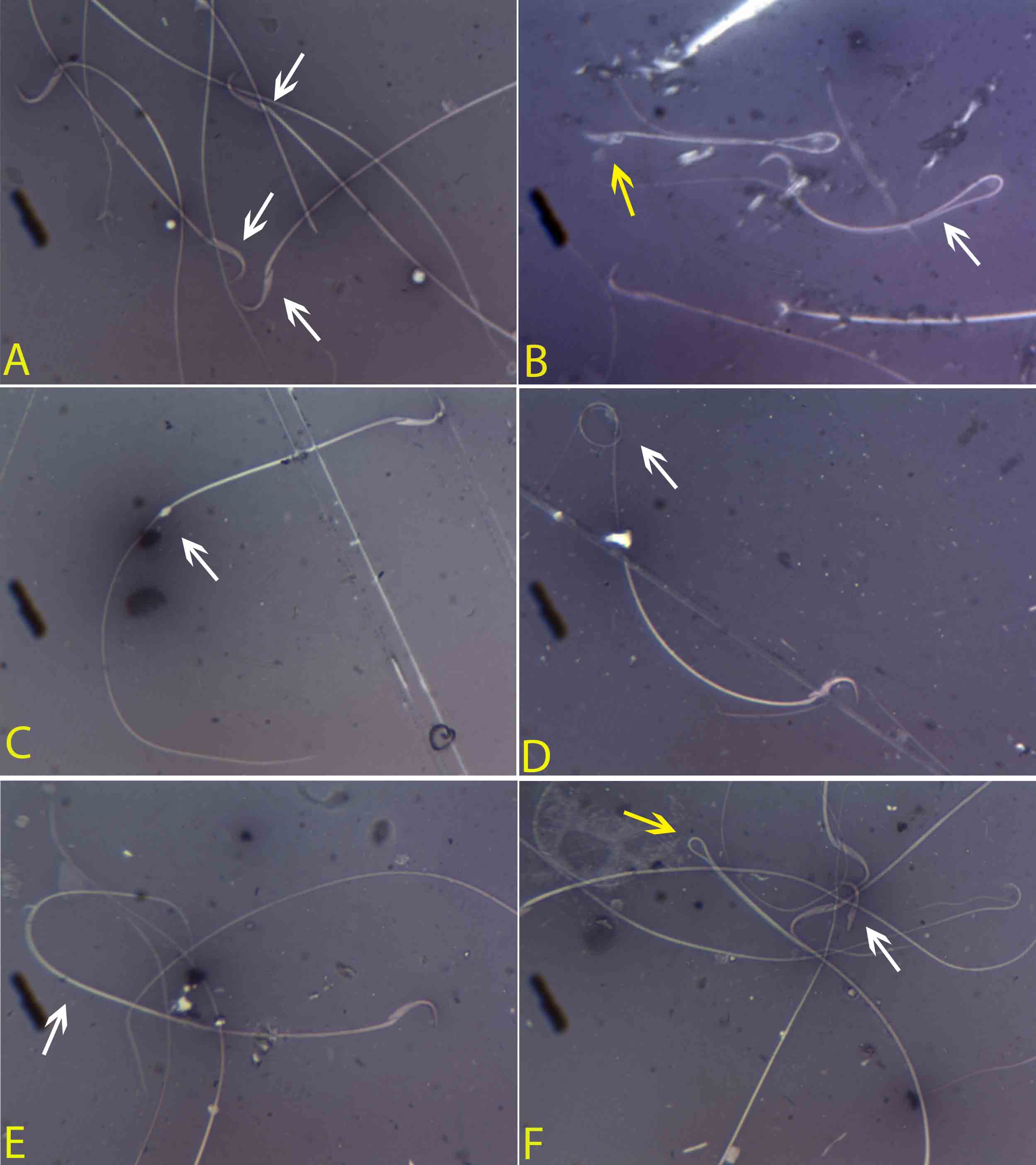


**Supplementary Figure 5**. Microphotographs illustrating morphologically normal sperm and various sperm defects. A – Normal sperm cells; B –Bent tail (White arrow) and amorphous head (Yellow arrow); C – Protoplasmic droplet; D – Coiled tail; E – Bent tail; F – Detached head (white arrow) and bent tail (Yellow arrow) (Eosin-nigrosine staining, Original magnification × 400).

**Supplementary Figure 6**. Effects of nano-particles of propolis and red ginseng either alone or in combination with dexamethasone on the semen vesicles glandular mRNA expression levels of CYP11, ARO, HSD-3b, StAR and Nrf-2 (Each bar represents mean ± SE).

-SE: Standard error.

- Different superscripts within the parameter were different (p <0.05).

**
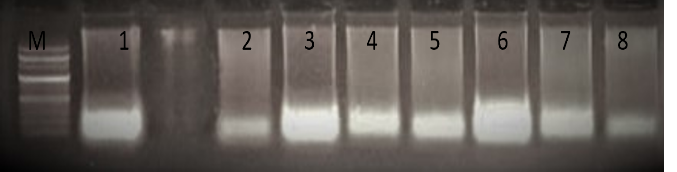
**

**
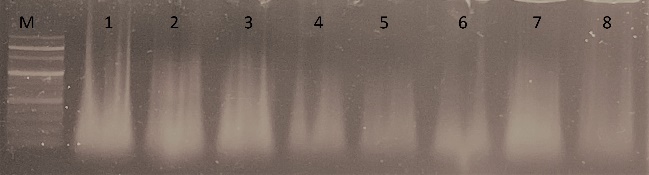
 A B**

**Supplementary Figure 7**. The electrophoresis of DNA fragments isolated from A: Testes; B: Seminal vesicle of treated rats. M represents the 100 bp DNA ladder, lane 1: CTRL-VE; lane 2: CTRL+VE; lane 3: Nano propolis + Dexamethasone; lane 4: Nano propolis; lane 5: Nano ginseng; lane 6: Nano ginseng+ dexamethasone; lane 7: propolis + Dexamethasone; lane8: Ginseng + Dexamethasone.


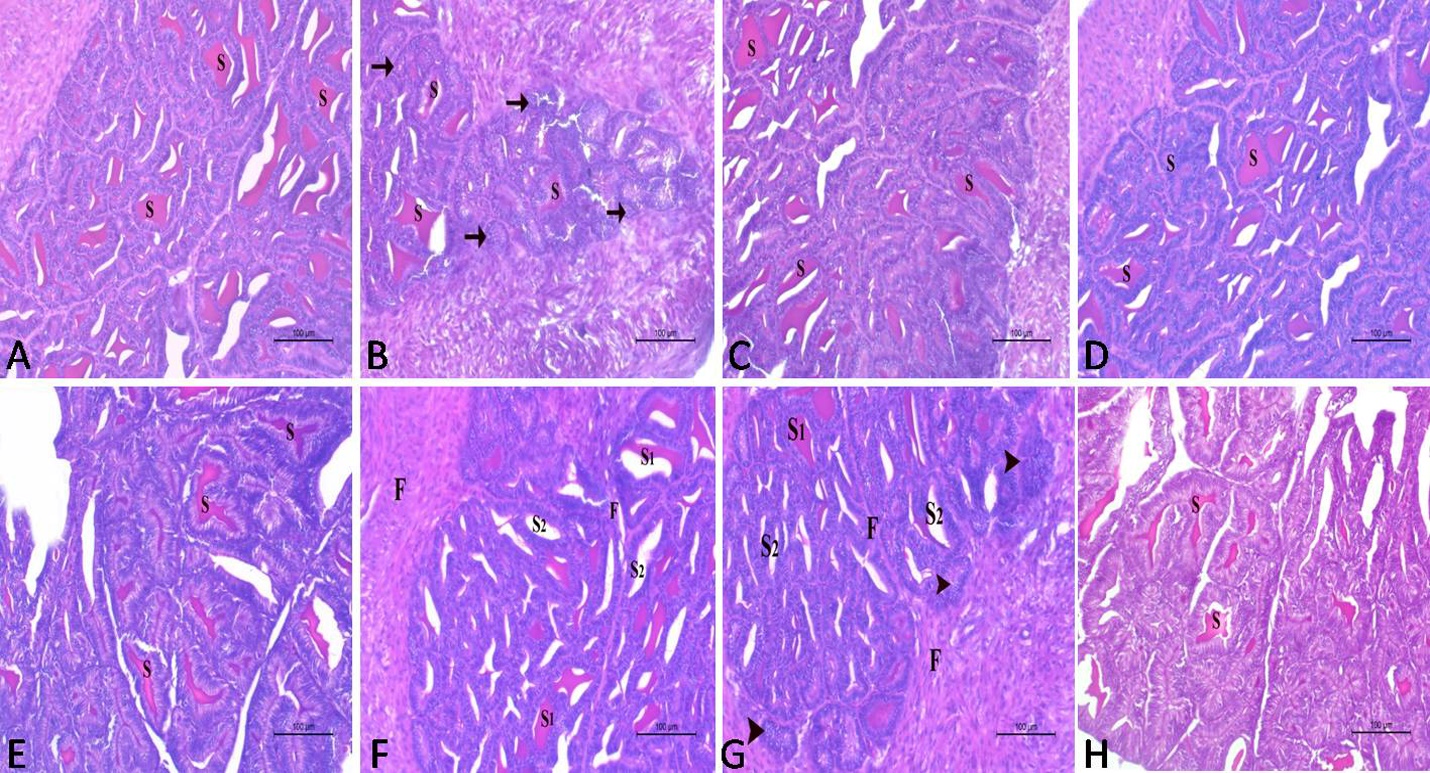


**Supplementary Figure 8.** A histological section in the vesicular gland of adult male albino rats stained with H&E X200.

A): Control group contained glands with normal secretory acini that were lined with high columnar epithelium detected in different stages of secretory activity. The lumen of the acini contained huge amounts of secretory materials (S).

B): Dexamethasone- treated group showed small, collapsed and inactive gland acini (arrow). The acini were lined with low columnar epithelium with little secretory activities. The interstitial connective tissue was highly proliferative and abundant around the acini. Note, few secretory materials appeared in the lumen of the acini (S).

C): Nano-propolis only group showed normal gland acini lined with high columnar cells that displayed different degrees of secretory activity. The acinar lumen contained a huge amount of secretory materials (S).

D): Nano-ginseng only group showed normal active acini of the vesicular gland. Note, secretory materials appeared in the lumen of the acini (S) and the other acini appeared exhausted.

E): Nano-propolis + dexamethasone group contained normal secretory acini lined by high columnar epithelium with secretory activity. The lumen of the acini contained huge amounts of secretory materials (S).

F): Nano-ginseng + dexamethasone-treated group showed normal acini with columnar epithelium displaying secretory activity and containing an adequate amount of secretory materials (S1). Other acini (S2) appeared less active without any secretory materials. The interstitial connective tissue (F) became proliferative and concentrated around and between the acini.

G): Propolis + dexamethasone group showed normal active acini with columnar epithelium and contained an adequate amount of secretory materials (S1). Other acini (S2) appeared less active without any secretory materials. The interstitial connective tissue (F) became proliferative and concentrated around and between the acini. Note, collapsed acini (arrow head) appeared in the gland. (H&E) stain X200.

H): Ginseng + dexamethasone group showed normal acini lined with high columnar epithelium present in several stages of secretion. The lumen of the acini was obliterated with huge amounts of secretory materials (S).

**Supplementary Table 1: Zeta potential and hydrodynamic size of propolis, red ginseng and their nano-preparations.**

| **Material** | **Zeta potential (mV)** | **Hydrodynamic Size(nm)** |
| --- | --- | --- |
| Propolis | -32.00±0.02 | 116±0.03 |
| Nano-propolis | -33.00±0.01 | 594±0.05 |
| Ginseng | -21.00±0.04 | 463±0.01 |
| Nano-ginseng | -18.00±0.01 | 725±0.02 |
